# Results from a phase 1, randomized, double-blind, multiple ascending dose study characterizing the pharmacokinetics and demonstrating the safety and selectivity of the aldosterone synthase inhibitor baxdrostat in healthy volunteers

**DOI:** 10.1038/s41440-022-01070-4

**Published:** 2022-10-20

**Authors:** Mason W. Freeman, Mary Bond, Brian Murphy, James Hui, Jonathan Isaacsohn

**Affiliations:** 1 CinCor Pharma, Inc., Waltham, MA USA; 2 CinRx Pharma, LLC, Cincinnati, OH USA

**Keywords:** Aldosterone synthase inhibitor, Aldosterone, CYP11B2, Hypertension, Primary aldosteronism

## Abstract

Baxdrostat is a selective inhibitor of aldosterone synthase designed for the treatment of disorders associated with elevated aldosterone. This study evaluated the safety, pharmacokinetics, and pharmacodynamics of multiple ascending doses of baxdrostat in healthy volunteers. Subjects were randomized to receive oral baxdrostat (0.5, 1.5, 2.5, or 5.0 mg) or placebo once daily for 10 days and were placed on either a low-salt or normal-salt diet for the duration of the study. Blood samples were collected before and after dosing on days 1 and 10 to characterize pharmacokinetics and pharmacodynamics. Safety was assessed by adverse events, physical examinations, electrocardiograms, orthostatic vital signs, and clinical laboratory evaluations. Fifty-four subjects completed the study. There were no deaths or serious adverse events, and all treatment-emergent adverse events in subjects receiving baxdrostat were mild in severity. Plasma levels of baxdrostat increased proportionally with ascending doses, with peak concentrations observed within 4 h after dosing and a mean half-life of 26 to 31 h. A dose-dependent reduction of plasma aldosterone occurred with baxdrostat doses ≥1.5 mg, regardless of diet. Decreases in plasma aldosterone were sustained, with levels reduced by approximately 51 to 73% on day 10. Baxdrostat had no meaningful impact on plasma cortisol levels and resulted in mild dose-dependent decreases in plasma sodium levels and increases in potassium levels. Baxdrostat was safe and well tolerated with a half-life that supports once-daily dosing. The dose-dependent reduction in plasma aldosterone and lack of effect on cortisol demonstrate the selective blockade of aldosterone synthase.

## Abstract (web summary)

This multiple ascending dose study of the selective aldosterone synthase inhibitor baxdrostat demonstrated a dose-dependent reduction in plasma aldosterone with no meaningful effect on plasma cortisol. There were no deaths or serious adverse events, and all treatment-emergent adverse events in subjects receiving baxdrostat were mild in severity.

## Graphical abstract

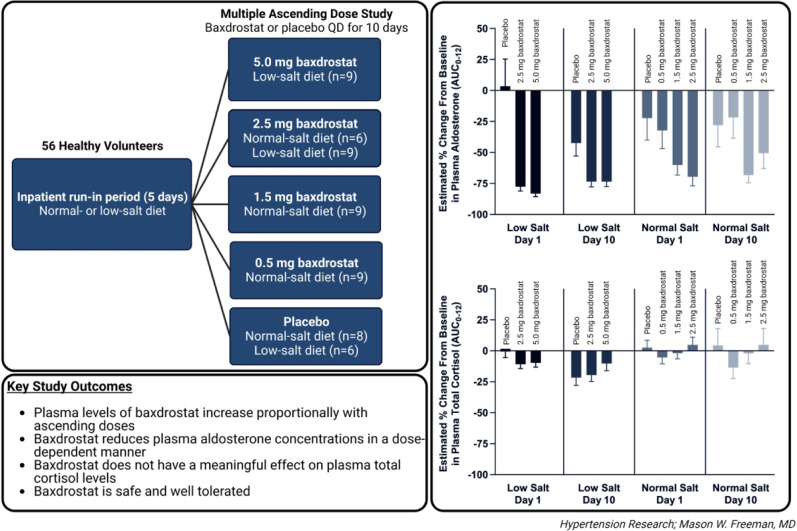

## Introduction

Aldosterone is a key component of the renin-angiotensin-aldosterone system (RAAS). It is produced in the zona glomerulosa of the adrenal cortex in response to angiotensin II, high extracellular potassium concentration, and adrenocorticotropic hormone (ACTH). Aldosterone is involved in the regulation of fluid and electrolyte homeostasis via activation of the mineralocorticoid receptor (MR) on various tissues, which causes vasoconstriction of vascular smooth muscle and increased water and sodium absorption by the kidneys. The rate-limiting enzyme in the synthetic pathway of aldosterone is aldosterone synthase (also known as CYB11B2).

Current first-line treatments for hypertension include diuretics, angiotensin-converting enzyme (ACE) inhibitors, and/or angiotensin receptor blockers (ARBs) [1]. However, aldosterone breakthrough can occur with long-term treatment with ACE inhibitors and ARBs [2]. The preferred agents for treating primary aldosteronism and treatment-resistant hypertension are MR antagonists (spironolactone and eplerenone) [1, 3], although long-term use of spironolactone is associated with a greater risk of gynecomastia and sexual dysfunction due to off-target effects [1, 4]. Eplerenone does not have these same side effects but is less potent than spironolactone [5].

Chronically elevated aldosterone levels can lead to hypokalemia, sodium reabsorption, and fluid retention, resulting in increased blood pressure (BP). Furthermore, inflammation, end organ damage, fibrosis, cardiovascular events (e.g., ventricular hypertrophy), and adverse renal events (e.g., increased urinary albumin excretion, progression of renal failure) can occur with increased aldosterone levels independently of BP [6]. The association between high plasma aldosterone and decreased long-term survival has been demonstrated in patients with congestive heart failure [7–10], acute myocardial infarction (MI) [11], and coronary artery disease outside the setting of acute MI or heart failure [12]. Thus, aldosterone synthase inhibition may be a promising therapeutic strategy for BP control and mitigation of end organ damage [13].

Baxdrostat (CIN-107) is a highly potent, selective, and competitive small molecule inhibitor of aldosterone synthase, as demonstrated by preclinical and first-in-human clinical studies [14, 15]. In vitro, baxdrostat exhibited a high selectivity ratio for aldosterone synthase compared to the highly homologous enzyme responsible for cortisol synthesis 11β-hydroxylase (CYP11B1) [15]. In vivo, nonhuman primate pharmacology characterization showed that baxdrostat blunted aldosterone production while having no effect on cortisol levels [15].

In accordance with the in vitro and in vivo studies, baxdrostat demonstrated selectivity for aldosterone synthase as shown by a dose-dependent reduction in plasma aldosterone with no effect on ACTH-stimulated plasma cortisol levels following administration of single ascending doses in the first-in-human study [15]. Although baxdrostat is the parent compound, multiple metabolites are present at lower concentrations. The active metabolites of baxdrostat also exhibited a high degree of selectivity for aldosterone synthase over 11β-hydroxylase. The primary metabolite, CIN-107-M, is a chiral molecule. The 2 enantiomers of CIN-107-M showed >20-fold greater selectivity for aldosterone synthase compared to 11β-hydroxylase; however, the more potent *R* enantiomer of CIN-107-M appears to not be formed in humans. Given the lower concentrations of the metabolites, the parent compound is thought to be the primary contributor to the pharmacologic effect (data on file). Baxdrostat had a favorable pharmacokinetic (PK) profile in humans. Plasma exposures increased in a dose-proportional manner over the expected therapeutic dose range with a mean half-life of 29 h, a result that supports once-daily dosing. Side effects were mild and included headache, nasopharyngitis, and diarrhea.

The favorable preclinical and early-phase clinical profiles of baxdrostat warranted assessment at steady state to confirm whether the effects are maintained following multiple doses [15], as this has not been the case for 2 previously explored aldosterone synthase inhibitors. In multiple-dose studies, LCI699 (Novartis Pharmaceuticals) was less selective for aldosterone synthase than for 11β-hydroxylase. Significant increases in the aldosterone precursor 11-deoxycorticosterone; dose-dependent accumulation of 11-deoxycortisol; and impairment of ACTH-stimulated cortisol synthesis indicated a latent inhibition of cortisol synthesis by LCI699 [16]. The potency of another aldosterone synthase inhibitor, LY3045697 (Eli Lilly and Company), was found to decrease significantly with daily dosing. The authors suggest that the decrease in potency could not be explained by elevated aldosterone precursors, but rather likely by an increase in aldosterone synthase activity over time [17].

These results support the importance of the current multiple ascending dose assessment to further the understanding of baxdrostat attained with the prior single-dose study [15]. This randomized, double-blind, placebo-controlled phase 1 study evaluated the safety, PK, and pharmacodynamics (PD) of multiple ascending doses of baxdrostat in healthy volunteers.

## Methods

### Study objectives

The primary objectives of this study were to assess safety, tolerability, PK, and PD of baxdrostat following oral dosing once daily for 10 days in subjects with a normal- or low-salt diet. The low-salt diet cohorts were included to stimulate aldosterone production and to evaluate safety in potential patients who may be following a low-salt diet.

### Subjects

Subjects were included in the study if they were between the ages of 18 and 55 years and in good health based on medical and psychiatric history, physical examination, electrocardiogram (ECG), orthostatic vital signs, and routine laboratory tests (blood chemistry, hematology, coagulation, and urinalysis). Subjects had to be nonsmokers and have a body mass index (BMI) ≥ 18 and ≤30 kg/m^2^. In addition, they were required to have an appropriate response to cortisol stimulation (cohorts 1 and 2) or a normal cortisol level during the inpatient run-in period (cohorts 3–5).

Subjects were excluded if they had (1) a personal or family history of long QT syndrome, complex ventricular arrythmias, or family history of sudden death; (2) personal history of or current clinically significant arrhythmias; (3) prolonged QTcF (>450 ms); (4) seated BP > 150/90 mm Hg or <90/50 mm Hg; (5) resting heart rate >100 beats per minute (bpm) or <50 bpm, sinus node dysfunction, or clinically significant heart block; (6) postural tachycardia or orthostatic hypotension; or (7) serum potassium greater than the upper limit of normal of the reference range and serum sodium less than the lower limit of normal of the reference range.

### Study design

Subjects were randomized in a 3:1 ratio to baxdrostat or placebo once daily for 10 days as follows:

- Cohort 1: 2.5 mg baxdrostat or matching placebo on a low-salt diet, 9 subjects receiving baxdrostat and 3 subjects receiving placebo
- Cohort 2: 5.0 mg baxdrostat or matching placebo on a low-salt diet, 9 subjects receiving baxdrostat and 3 subjects receiving placebo
- Cohort 3: 1.5 mg baxdrostat or matching placebo on a normal-salt diet, 9 subjects receiving baxdrostat and 3 subjects receiving placebo
- Cohort 4: 2.5 mg baxdrostat or matching placebo on a normal-salt diet, 6 subjects receiving baxdrostat and 2 subjects receiving placebo
- Cohort 5: 0.5 mg baxdrostat or matching placebo on a normal-salt diet, 9 subjects receiving baxdrostat and 3 subjects receiving placebo.

Cohorts 1 and 2 were dosed concurrently with a minimum 5-day lag between the first dose for cohort 2 and the first dose for cohort 1. A data review committee met between cohorts 2 and 3 and determined that cohorts 3 through 5 could be dosed concurrently. Each patient had a screening period of up to 28 days, followed by a 5-day run-in period in which the subjects adhered to a controlled, standardized diet and underwent baseline PD assessments (Supplementary Fig. 1). This was followed by a 15-day inpatient treatment period in which the subjects received baxdrostat or placebo once daily for 10 days while remaining on the controlled, standardized diet. Blood and urine sample collection for PK and PD analysis continued for an additional 5 days and was proceeded by a follow-up visit 3 ± 1 days after clinic discharge.

The study drug was a 2 mg/mL baxdrostat oral solution and matching oral placebo solution. Subjects received a single dose of oral solution of baxdrostat or placebo on the mornings of days 1 through 10 at 8:00 a.m. (± 2 h). On days 1 and 10, the dose was given following an overnight fast (of at least 8 h), and subjects remained fasted for at least 4 h after dosing on these days. On all other days, subjects fasted for a minimum of 2 h before and 2 h after dosing.

For cohort 1, the controlled, standardized low-salt diet consisted of 50 to 60 mEq Na^+^/d and 70 to 100 mEq K^+^/d from day −5 until day −1. Based on decreases in sodium levels observed in some subjects during the run-in period prior to any subjects receiving the study drug, the diet was modified to 65 to 70 mEq Na^+^/d and 70 to 100 mEq K^+^/d from day 1 through the remainder of the study. Cohort 2 subjects were given a low-salt diet consisting of up to 65 to 70 mEq Na^+^/d and 70 to 100 mEq K^+^/d for the full run-in and treatment periods. Cohorts 3 through 5 were on a normal-salt diet of 100 to 104 mEq Na^+^/d and 70 to 100 mEq K^+^/d for the full run-in and treatment periods.

Cohorts 1 and 2 also underwent an ACTH (Cortrosyn^TM^) challenge at baseline (day −1), 1 h after the first dose of study drug on day 1, and 1 h after the final dose of study drug on day 10. This was done to increase aldosterone and cortisol levels to more thoroughly evaluate the specificity of baxdrostat for targeting aldosterone synthase. The ACTH challenge consisted of an intravenous injection of 0.25 mg Cortrosyn^TM^ administered over 2 min after an overnight fast.

### Analytical methods

Plasma and urine samples were analyzed to measure concentrations of baxdrostat and its primary metabolite (CIN-107-M), aldosterone and its precursors, cortisol and its precursor, and ACTH using a validated liquid chromatography tandem mass spectrometry method (LC-MS/MS). The quantifiable ranges for both baxdrostat and its metabolite CIN-107-M in plasma and urine were 0.05 to 50 ng/mL (low range) and 5 to 2500 ng/mL (high range), using baxdrostat-d_5_ and CIN-107-M-d_3_, respectively, as the internal standards. Plasma samples were extracted by protein precipitation with methanol, followed by analysis by LC-MS/MS with electrospray ionization in positive mode and multiple reaction monitoring. All scheduled study-related laboratory tests were performed by Medpace Reference Laboratories (Cincinnati, OH, USA), Medpace Bioanalytical Laboratories (Cincinnati, OH, USA), Mercy Health—West Hospital Laboratory (Cincinnati, OH, USA), or Laboratory Corporation of America (Dublin, OH, USA).

### Pharmacokinetic analyses

Blood samples were collected prior to and after dosing on days 1 and 10 for measurement of plasma baxdrostat and CIN-107-M concentrations to characterize single-dose and steady-state PK. Pharmacokinetic parameters were calculated by standard noncompartmental methods using Phoenix WinNonlin™ (Certara, Inc., Princeton, NJ) and verified with SAS (SAS Institute, Inc., Cary, NC) software. Plasma PK parameters included maximum observed plasma concentration on day 1 (C_max,D1_) and day 10 (C_max,D10_), time to C_max_ on day 1 (T_max,D1_) and day 10 (T_max,D10_), and area under the plasma concentration-time curve (AUC) from time 0 to 24 h postdose on day 1 (AUC_0-24 h_) or from time 0 to 24 h on day 10 (AUC_0-tau_). Steady-state plasma PK parameters consisted of accumulation ratio based on C_max_ (R_Cmax_), accumulation ratio based on AUC (R_AUC_), apparent first-order terminal elimination rate constant (λ_z_), terminal phase elimination half-life (t_1/2_), apparent plasma clearance (CL_ss_/F), and apparent volume of distribution (V_ss_/F). An exploratory dose proportionality analysis of baxdrostat, C_max,D1_, and AUC_0-24 h_ (day 1) or C_max,D10_, AUC_0-tau_, and AUC_0-t_ (day 10) was performed using a power model to estimate the mean slope and 90% confidence interval (CI).

The cumulative amount of drug excreted in urine on day 1 (A_e,D1_) and day 10 (A_e,D10_), fraction of dose excreted renally on day 1 (F_e,D1_) and day 10 (F_e,D10_), and renal clearance on day 1 (CL_R,D1_) and day 10 (CL_R,D10_) composed single-dose urine PK parameters.

### Pharmacodynamic and safety analyses

Plasma PD measures included concentrations of aldosterone, 18-hydroxycorticosterone, corticosterone, 11-deoxycorticosterone, cortisol (free and total), 11-deoxycortisol, ACTH, sodium, chloride, and potassium. AUCs were calculated using a noncompartmental analysis and the linear trapezoidal/linear interpolation method. Urine PD measures included concentrations of aldosterone, cortisol (free and total), tetrahydroaldosterone, sodium, chloride, potassium, creatinine, and phosphorus. Other PD measures were seated, orthostatic, and positional changes in vital signs, as well as changes in body weight and BMI. Safety assessments involved adverse events, physical examinations, ECGs, orthostatic vital signs, and clinical laboratory evaluations.

### Statistical analyses

Subjects who received placebo were pooled into 2 treatment groups: placebo with low-salt diet and placebo with normal-salt diet. Descriptive statistics were calculated for PK, PD, and safety measures. The effects of dose on plasma aldosterone and cortisol (free and total) and their precursors were investigated using an analysis of variance model on change in AUC over 12 h from day −1 to day 1 and from day −1 to day 10. Data are presented as change from baseline in least squares mean (90% CI) AUC_0-12h_ of plasma aldosterone on days 1 and 10. The results of the exploratory dose proportionality power model were assessed using the Smith criteria (0.9031, 1.0969) [18] and the less stringent Hummel criteria (0.6990, 1.3010) [19].

Three subjects had negligible aldosterone concentrations on day −1 that increased to more expected levels on days 1 and 10. Including these subjects in the baseline analyses skewed the results and disrupted cross-treatment comparisons; therefore, they were considered outliers and were not included in the data presented here.

## Results

### Subjects

Demographics and baseline characteristics are presented in Table 1. Most study participants were male (70%), Black or African American (50%), and not Hispanic or Latino (88%). The average age was 40 years, and mean BMI ranged from 24 to 27 kg/m^2^. The demographic and baseline characteristics were generally well matched in sex and race across treatment groups.

**Table 1:** Baseline demographics and clinical characteristics

Demographic or characteristic	Low-salt diet			Normal-salt diet			
	Pooled placebo (*n* = 6)	2.5 mg baxdrostat (*n* = 9)	5.0 mg baxdrostat (*n* = 9)	Pooled placebo (*n* = 8)	0.5 mg baxdrostat (*n* = 9)	1.5 mg baxdrostat (*n* = 9)	2.5 mg baxdrostat (*n* = 6)
Age (y), mean ± SD	43.8 ± 6.4	37.2 ± 8.7	39.3 ± 10.2	37.0 ± 8.7	37.9 ± 8.6	44.8 ± 8.6	39.0 ± 9.7
Race, *n* (%)							
White	2 (33.3)	4 (44.4)	5 (55.6)	5 (62.5)	3 (33.3)	3 (33.3)	4 (66.7)
Black or African American	4 (66.7)	5 (55.6)	3 (33.3)	3 (37.5)	6 (66.7)	5 (55.6)	2 (33.3)
Asian	0 (0.0)	0 (0.0)	0 (0.0)	0 (0.0)	0 (0.0)	1 (11.1)	0 (0.0)
Other	0 (0.0)	0 (0.0)	1 (11.1)	0 (0.0)	0 (0.0)	0 (0.0)	0 (0.0)
Ethnicity, *n* (%)							
Hispanic or Latino	1 (16.7)	0 (0.0)	2 (22.2)	1 (12.5)	2 (22.2)	1 (11.1)	0 (0.0)
Not Hispanic or Latino	5 (83.3)	9 (100.0)	7 (77.8)	7 (87.5)	7 (77.8)	8 (88.9)	6 (100.0)
Sex, *n* (%)							
Female	1 (16.7)	4 (44.4)	3 (33.3)	1 (12.5)	4 (44.4)	1 (11.1)	3 (50.0)
Male	5 (83.3)	5 (55.6)	6 (66.7)	7 (87.5)	5 (55.6)	8 (88.9)	3 (50.0)
Height (cm), mean ± SD	175.3 ± 10.2	168.9 ± 10.7	170.1 ± 12.2	170.2 ± 8.6	171.5 ± 11.5	173.4 ± 9.7	171.7 ± 8.2
Body weight (kg), mean ± SD	78.3 ± 13.2	77.0 ± 11.0	75.8 ± 12.0	70.4 ± 11.0	79.5 ± 12.0	78.6 ± 11.3	74.9 ± 5.5
BMI (kg/m^2^), mean ± SD	25.3 ± 2.5	26.9 ± 1.4	26.0 ± 1.7	24.3 ± 3.2	26.9 ± 1.8	26.0 ± 2.5	25.5 ± 2.4

*BMI* body mass index

### Pharmacokinetics

**Fig. 1:**
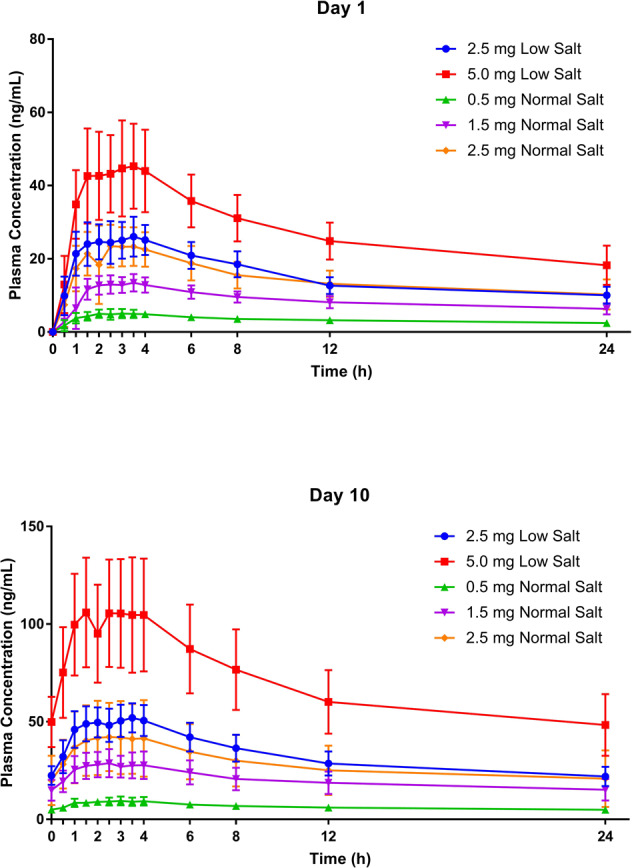
Plasma Baxdrostat Concentration vs. Time on Days 1 and 10. Plasma baxdrostat concentration (ng/mL) by scheduled time point and treatment. Data are mean ± standard deviation

Single-dose and steady-state plasma PK parameters of baxdrostat are presented in Supplementary Table 1. Baxdrostat was rapidly absorbed with a median T_max_ observed within 4 h of dosing (Fig. 1). Baxdrostat concentrations declined from peak in an apparent biphasic manner with a mean t_1/2_ ranging from approximately 26 to 31 h. The plasma concentration-time profile of baxdrostat demonstrated increased exposure with increasing dose. At steady state, baxdrostat exposure was approximately 2- to 2.5-fold higher than after a single dose.

An exploratory analysis to assess dose proportionality demonstrated that with increasing doses of baxdrostat there was a proportional increase in both C_max_ and AUC_0-24 h_ on day 1 (according to the Hummel criteria). Similarly, proportional increases in C_max_, AUC_0-tau_, and AUC_0-t_ on day 10 were also observed.

Although CIN-107-M is not believed to contribute substantially to the effects of baxdrostat, its prevalence warrants characterization. On average, CIN-107-M represents 8 to 11% of the parent compound based on C_max,D10_ and 10 to 22% of the parent compound based on AUC_0-inf_. Based on plasma concentration data, CIN-107-M was formed ~4 to 24 h after the initial dose of baxdrostat on day 1 and was typically observed within 4 h at steady state (day 10). Plasma levels of CIN-107-M showed a dose-proportional increase similar to the PK of the parent compound (data not shown). Approximately 7% (mean F_e,D1_ ranged from 6.3 to 10.8% across treatment groups) of the baxdrostat dose was recovered unchanged in the urine on day 1. At steady state (day 10), ~32% was recovered unchanged (mean F_e,D10_ ranged from 30.7 to 33.6% across treatment groups).

### Pharmacodynamics

**Fig. 2:**
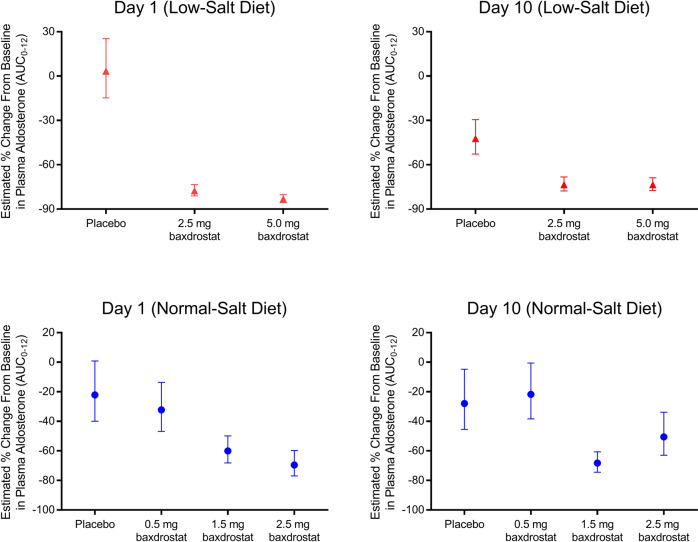
Baxdrostat Reduces Plasma Aldosterone Levels in a Dose-dependent Manner. Estimated percentage change from baseline (day −1) in plasma aldosterone area under the pharmacodynamic effect-time curve from time 0 to 12 h postdose on day 1 and day 10. Data are least squares mean and 90% confidence intervals. AUC indicates area under the curve

A dose-dependent reduction of plasma aldosterone (estimated percentage change from baseline AUC_0-12h_) occurred with baxdrostat doses ≥1.5 mg, regardless of normal- or low-salt diet. Decreases in plasma aldosterone were observed starting on day 1 and were sustained, with levels reduced by ~51 to 73% on day 10 (Fig. 2). Corresponding dose-dependent decreases of aldosterone and tetrahydroaldosterone urine excretion were also observed (data not shown).

Baxdrostat caused a modest increase in the aldosterone precursor 11-deoxycorticosterone under both normal- and low-salt diet conditions. Additional aldosterone precursors demonstrated stepwise changes indicative of the progressive impact of aldosterone synthase inhibition on the pathway of aldosterone synthesis. 18-hydroxycorticosterone (the immediate precursor to aldosterone) levels were generally comparable or decreased compared to baseline, although to a lesser extent than aldosterone. Corticosterone levels increased in an apparent dose-dependent manner, likely due to 11β-hydroxylase activity (Supplementary Fig. 2).

**Fig. 3:**
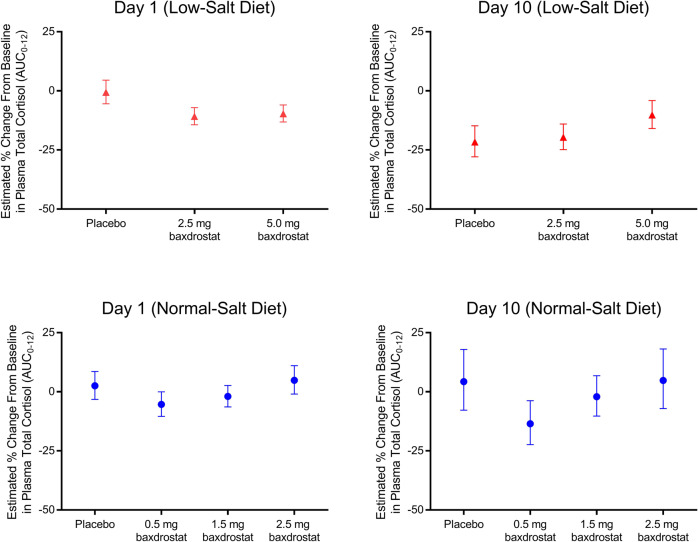
Baxdrostat Does Not Have a Significant Effect on Plasma Cortisol Levels. Estimated percentage change from baseline (day −1) in plasma cortisol (total) area under the pharmacodynamic effect-time curve from time 0 to 12 h postdose on day 1 and day 10. Data are least squares mean and 90% confidence intervals. AUC indicates area under the curve

Baxdrostat had no meaningful effect on plasma cortisol (total or free) or 11-deoxycortisol concentrations even in the presence of the ACTH challenge in the low-salt diet groups (Fig. 3). Likewise, there was no meaningful effect on urine free cortisol (Supplementary Fig. 3). Subjects on a low-salt diet had increased plasma ACTH levels at baseline. The increases were somewhat more pronounced in subjects receiving baxdrostat compared to placebo. Under normal-salt conditions, however, baxdrostat resulted in a dose-dependent decrease in ACTH.

There were no clinically significant changes or dose-related trends in seated heart rate or BP (Supplementary Fig. 4). A slight trend toward mild, drug-induced decreases in orthostatic BP and moderate increases in orthostatic heart rate were observed, but there was no clear dose-dependency. The most pronounced effects on heart rate occurred in the 5-mg baxdrostat treatment group.

**Fig. 4:**
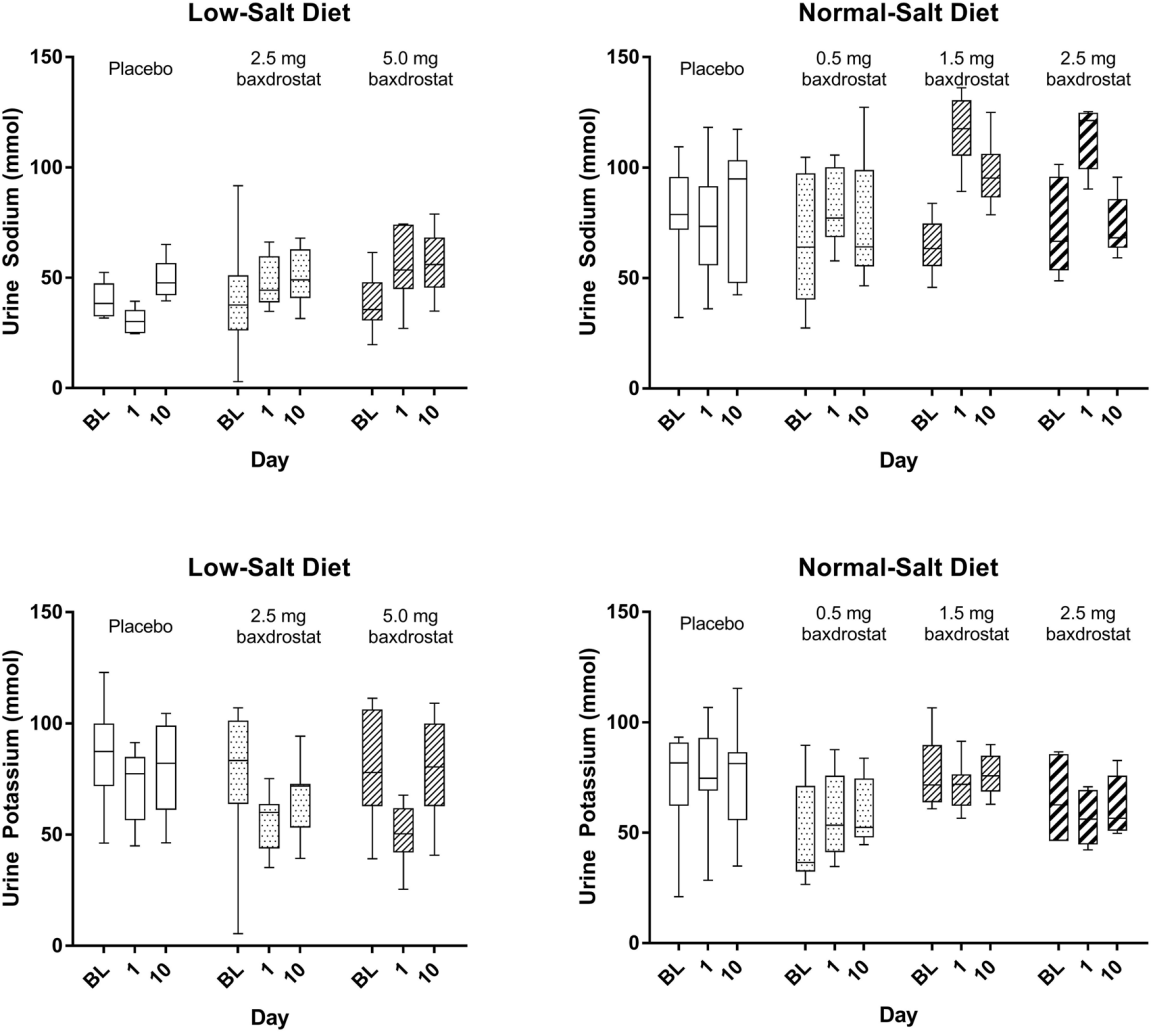
Urinary Sodium and Potassium Concentrations Following Baxdrostat Administration. Urine sodium and potassium concentrations at baseline (BL), day 1, and day 10. Box whiskers show interquartile range (box) and minimum-maximum (whiskers) for sodium or potassium from 24 h urine collections

Baxdrostat administration resulted in mild dose-dependent decreases in plasma sodium levels and increases in potassium levels, as would be expected from the observed reduction in aldosterone (Supplementary Fig. 5). Urine sodium and potassium levels corresponded to the changes observed in plasma (increased sodium, decreased potassium). The sodium:potassium ratio was increased (sodium loss in the urine was greater than the potassium retention) on day 1 following the first dose of baxdrostat; however, this effect was diminished by day 10. This appears to be mediated by a greater elimination of sodium on day 1 compared to day 10, as potassium did not substantially change over the course of the treatment period (Fig. 4).

Renal function was assessed by plasma blood urea nitrogen (BUN), creatinine, and glomerular filtration rate. There were mild increases in BUN, creatinine, and the BUN:creatinine ratio. A mild reduction in glomerular filtration rate (<15%) was also observed. These results suggests that baxdrostat has a mild diuretic effect. Mean body weight and BMI decreased slightly from baseline during the treatment period in all treatment groups, including placebo; however, the decrease was more pronounced in subjects receiving baxdrostat. The values largely returned to baseline at the follow-up visit.

There were no deaths, serious adverse events, or discontinuations due to treatment-emergent adverse events (TEAEs). Overall, 6 (14.3%) subjects receiving baxdrostat and 3 (21.4%) subjects receiving placebo experienced a TEAE that was considered related to the study drug. All TEAEs in subjects receiving baxdrostat were mild (Table 2). There were no clinically meaningful changes in physical examination findings, vital signs, electrocardiograms (including no QT prolongation), or clinical laboratory results related to safety.

**Table 2:** Summary of adverse events

Adverse events, *n* (%)	Low-salt diet			Normal-salt diet			
	Pooled placebo (*n* = 6)	2.5 mg baxdrostat (*n* = 9)	5.0 mg baxdrostat (*n* = 9)	Pooled placebo (*n* = 8)	0.5 mg baxdrostat (*n* = 9)	1.5 mg baxdrostat (*n* = 9)	2.5 mg baxdrostat (*n* = 6)
Any AE	2 (33.3)	3 (33.3)	3 (33.3)	1 (12.5)	1 (11.1)	1 (11.1)	3 (50.0)
Any TEAE	2 (33.3)	3 (33.3)	3 (33.3)	1 (12.5)	1 (11.1)	1 (11.1)	3 (50.0)
Palpitations	0 (0.0)	0 (0.0)	0 (0.0)	1 (12.5)	0 (0.0)	0 (0.0)	0 (0.0)
Ventricular tachycardia	1 (16.7)	0 (0.0)	0 (0.0)	0 (0.0)	0 (0.0)	0 (0.0)	0 (0.0)
Eye irritation	0 (0.0)	0 (0.0)	0 (0.0)	0 (0.0)	0 (0.0)	0 (0.0)	1 (16.7)
Abdominal pain	0 (0.0)	0 (0.0)	1 (11.1)	0 (0.0)	0 (0.0)	0 (0.0)	0 (0.0)
Constipation	0 (0.0)	0 (0.0)	0 (0.0)	0 (0.0)	0 (0.0)	1 (11.1)	0 (0.0)
Nausea	1 (16.7)	0 (0.0)	0 (0.0)	0 (0.0)	0 (0.0)	0 (0.0)	0 (0.0)
Rhinitis	0 (0.0)	0 (0.0)	0 (0.0)	0 (0.0)	0 (0.0)	0 (0.0)	1 (16.7)
Viral infection	0 (0.0)	1 (11.1)	0 (0.0)	0 (0.0)	0 (0.0)	0 (0.0)	0 (0.0)
Back pain	0 (0.0)	0 (0.0)	1 (11.1)	0 (0.0)	0 (0.0)	0 (0.0)	0 (0.0)
Headache	0 (0.0)	1 (11.1)	1 (11.1)	0 (0.0)	1 (11.1)	0 (0.0)	1 (16.7)
Dizziness postural	0 (0.0)	1 (11.1)	0 (0.0)	0 (0.0)	0 (0.0)	0 (0.0)	2 (33.3)
Dizziness	0 (0.0)	0 (0.0)	1 (11.1)	0 (0.0)	0 (0.0)	0 (0.0)	1 (16.7)
Presyncope	0 (0.0)	1 (11.1)	0 (0.0)	0 (0.0)	0 (0.0)	0 (0.0)	0 (0.0)
Anxiety	0 (0.0)	0 (0.0)	0 (0.0)	0 (0.0)	0 (0.0)	0 (0.0)	1 (16.7)
Dry throat	0 (0.0)	0 (0.0)	0 (0.0)	0 (0.0)	0 (0.0)	0 (0.0)	1 (16.7)
Dysphonia	0 (0.0)	0 (0.0)	1 (11.1)	0 (0.0)	0 (0.0)	0 (0.0)	0 (0.0)
Any treatment-emergent SAE	0 (0.0)	0 (0.0)	0 (0.0)	0 (0.0)	0 (0.0)	0 (0.0)	0 (0.0)
Any drug-related treatment-emergent SAE	0 (0.0)	0 (0.0)	0 (0.0)	0 (0.0)	0 (0.0)	0 (0.0)	0 (0.0)
Any TEAE leading to death	0 (0.0)	0 (0.0)	0 (0.0)	0 (0.0)	0 (0.0)	0 (0.0)	0 (0.0)
Any TEAE leading to discontinuation	0 (0.0)	0 (0.0)	0 (0.0)	0 (0.0)	0 (0.0)	0 (0.0)	0 (0.0)

TEAEs are defined as any AE, regardless of relationship to the study drug, which began after the first dose was administered

*AE* adverse event, *SAE* serious adverse event, *TEAE* treatment-emergent adverse event

## Discussion

Consistent with the single ascending dose study results [15], oral dosing of baxdrostat resulted in dose-proportional increases in plasma baxdrostat and its metabolite with a relatively long half-life of 26 to 31 h, resulting in support of once-daily dosing.

One of the challenges of aldosterone synthase inhibition is to achieve high selectivity for the enzyme, which shares 93% homology to cortisol synthase [20]. Suppression of cortisol could lead to compromised stress response, impaired immune system, inadequate metabolism, and possibly increased mortality rates [21, 22]. Based on preclinical studies, it was predicted that baxdrostat would selectively inhibit aldosterone synthase [15]. Our results demonstrate that at doses ≥1.5 mg, baxdrostat produces a dose-dependent decrease in aldosterone compared to baseline and placebo while having no meaningful impact on cortisol levels, supporting the selective nature of baxdrostat. Importantly, the aldosterone-lowering effect was sustained, suggesting no apparent compensatory mechanisms over the duration of the study and thus differentiating baxdrostat from the previously studied aldosterone synthase inhibitors LCI699 and LY3045697.

Lack of specificity for aldosterone synthase has resulted in the discontinuation of LCI699, which was previously being developed for the treatment of hypertension [16]. LCI699 was selective for aldosterone synthase after a single dose; however, after multiple-dose administration it showed a time-dependent loss of selectivity [23]. LY3045697 lost its aldosterone-reducing potency after multiple doses (half maximal inhibitory concentration was 47 times higher than after single-dose administration) despite similar PK with both single- and multiple-dose administration [17].

Baxdrostat decreased plasma aldosterone levels under both low-salt and normal-salt diet conditions; the 2.5-mg baxdrostat dose used in both low- and normal-salt conditions showed a similar effect on lowering plasma aldosterone levels. The low-salt diet subjects of cohorts 1 and 2 also underwent an ACTH challenge to further stimulate aldosterone and cortisol levels prior to receiving baxdrostat. Baxdrostat suppressed plasma aldosterone levels while having no meaningful effect on cortisol, even in the presence of the ACTH challenge, further supporting the selectivity of baxdrostat to inhibit aldosterone synthase.

As expected, the inhibition of aldosterone synthase by baxdrostat resulted in an increase in the aldosterone precursor 11-deoxycorticosterone under both normal- and low-salt diet conditions. However, the magnitude of increase was modest (~2- to 3-fold) compared to what has previously been observed for LCI699, where 11-deoxycorticosterone levels increased up to 10-fold [24].

Although the study was conducted in healthy volunteers, the apparent diuretic effect, the slight trends toward mild baxdrostat-induced decreases in orthostatic BP, and the moderate increases in orthostatic heart rate indicate that baxdrostat represents a promising treatment for the negative health impact of elevated aldosterone. It is difficult to predict how the observed 51 to 73% reduction in plasma aldosterone will translate to BP reduction in patients with primary aldosteronism or uncontrolled or treatment-resistant hypertension; however, further studies in these patient populations will help elucidate this relationship.

There are potential benefits of selective aldosterone synthase inhibition vs. MR antagonism. For example, due to the hypothalamic-pituitary axis feedback loop, blocking MRs activates the RAAS, leading to a compensatory increase in aldosterone, which could overcome the blockade by MR antagonists [25]. In addition, current MR antagonists (spironolactone and eplerenone) are accompanied by adverse events, such as sexual dysfunction, gynecomastia in men, and electrolyte imbalances [26]. Baxdrostat was well tolerated with a favorable safety profile. The most common TEAEs in subjects receiving baxdrostat were headache, postural dizziness, and dizziness.

The relatively small sample size for each treatment group is a limitation of the current study. Because it was conducted in healthy subjects, the clinical relevance of these findings needs to be investigated in patients who have uncontrolled or treatment-resistant hypertension or primary aldosteronism. As expected, we were unable to detect BP changes in our healthy subjects and therefore were not able to definitively identify the efficacious dose range for patients with hypertension or primary aldosteronism. Moreover, the relationship between the magnitude of aldosterone lowering and BP lowering is not yet defined. For these reasons, further study is needed to understand the dose-response for lowering BP in these patients, including the possibility of additional higher doses.

## Conclusions

Oral administration of baxdrostat was safe and well tolerated in all subjects and resulted in dose-proportional increases in plasma baxdrostat with a half-life that supports once-daily dosing. The dose-dependent decrease in plasma aldosterone at doses ≥1.5 mg and lack of effect on cortisol demonstrate the selective blockade of aldosterone synthase and support continued study in ongoing phase 2 clinical trials evaluating the efficacy and safety of baxdrostat for treatment-resistant or uncontrolled hypertension and primary aldosteronism.

## Supplementary information

Supplementary Information
