# Supplementary material for: Results from a phase 1, randomized, double-blind, multiple ascending dose study characterizing the pharmacokinetics and demonstrating the safety and selectivity of the aldosterone synthase inhibitor baxdrostat in healthy volunteers
Source: Hypertens Res. 2022 Oct 20;46(1):108–18. doi: 10.1038/s41440-022-01070-4 (PMC9747611; doi:10.1038/s41440-022-01070-4)
Supplement: Supplementary file 1 — Supplementary Information [file 41440_2022_1070_MOESM1_ESM.docx]

# Supplementary Figures and Tables

| Supplementary Table 1. Summary of Single-Dose and Steady-State Plasma PK Parameters of Baxdrostat | | | | | |
| --- | --- | --- | --- | --- | --- |
| Plasma PK Parameter | **Low-salt diet** | | **Normal-salt diet** | | |
|  | **2.5 mg**  **baxdrostat**  **(n=9)** | **5.0 mg**  **baxdrostat**  **(n=9)** | **0.5 mg**  **baxdrostat**  **(n=9)** | **1.5 mg**  **baxdrostat**  **(n=9)** | **2.5 mg**  **baxdrostat**  **(n=6)*** |
| Day 1 | | | | | |
| C_max,D1_ (ng/mL) | 28.09 ± 5.90 | 47.33 ± 12.30 | 5.36 ± 1.25 | 14.03 ± 2.24 | 26.18 ± 4.76 |
| T_max,D1_ (h), median (min, max) | 3.00 (0.98, 4.00) | 3.02 (1.50, 4.00) | 2.00 (1.00, 4.00) | 2.50 (1.00, 4.00) | 3.00 (2.00, 4.00) |
| AUC_0-24hr_ (h‧ng/mL) | 365.79 ± 65.51 | 657.85 ± 137.40 | 79.47 ± 17.34 | 205.31 ± 36.74 | 343.98 ± 92.44 |
| Day 10 | | | | | |
| C_max,D10_ (ng/mL) | 53.96 ± 7.56 | 113.44 ± 25.86 | 9.72 ± 2.27 | 29.94 ± 6.10 | 43.88 ± 18.69 |
| T_max,D10_ (h), median (min, max) | 2.00 (0.98, 4.00) | 3.00 (1.50, 4.00) | 2.00 (1.00, 3.50) | 2.50 (1.00, 4.00) | 3.00 (2.50, 4.00) |
| AUC_0-tau_ (h‧ng/mL) | 782.99 ± 143.02 | 1659.41 ± 440.51 | 155.19 ± 35.70 | 479.67 ± 138.21 | 676.51 ± 343.78 |
| t_1/2_ (h) | 28.37 ± 4.75 | 29.36 ± 6.47 | 31.16 ± 5.80 | 29.92 ± 7.71 | 25.55 ± 5.95 |
| CL_ss_/F (L/h) | 3.28 ± 0.53 | 3.21 ± 0.84 | 3.42 ± 1.01 | 3.35 ± 0.92 | 4.32 ± 1.59 |
| V_ss_/F (L) | 134.70 ± 36.70 | 134.27 ± 41.98 | 151.58 ± 44.50 | 136.87 ± 15.77 | 150.38 ± 42.42 |
| R_Cmax_ | 1.97 ± 0.30 | 2.42 ± 0.33 | 1.83 ± 0.26 | 2.13 ± 0.24 | 1.71 ± 0.58 |
| R_AUC_ | 2.15 ± 0.16 | 2.51 ± 0.26 | 1.96 ± 0.27 | 2.31 ± 0.32 | 1.99 ± 0.45 |
| *n=5 for day 10 measurements.  Data are mean ± standard deviation unless otherwise indicated.  Abbreviations: AUC_0-24h_, area under the plasma concentration-time curve from time 0 to 24 hours postdose; AUC_0-tau_, area under the plasma concentration-time curve over a dosing interval; CL_ss_/F, apparent plasma clearance; C_max,D1_, maximum observed plasma concentration on day 1; C_max,D10_, maximum observed plasma concentration on day 10; max, maximum; min, minimum; PK, pharmacokinetics; R_AUC_, accumulation ratio based on the area under the plasma concentration-time curve after the first dose and the last dose; R_Cmax_, accumulation ratio based on the maximum observed plasma concentration after first dose and the final dose; t_½_, terminal phase elimination half-life; T_max_, time to maximum observed plasma concentration; V_ss_/F, apparent volume of distribution. Lower limit of quantitation of baxdrostat = 0.05 ng/mL. | | | | | |


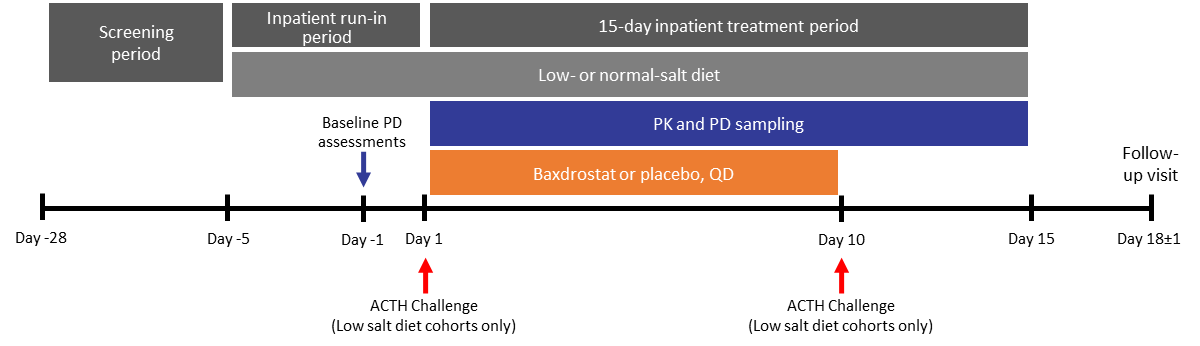


**Supplementary Figure 1. Study Design**

ACTH indicates adrenocorticotropic hormone; PD, pharmacodynamic; PK, pharmacokinetic; QD, daily.


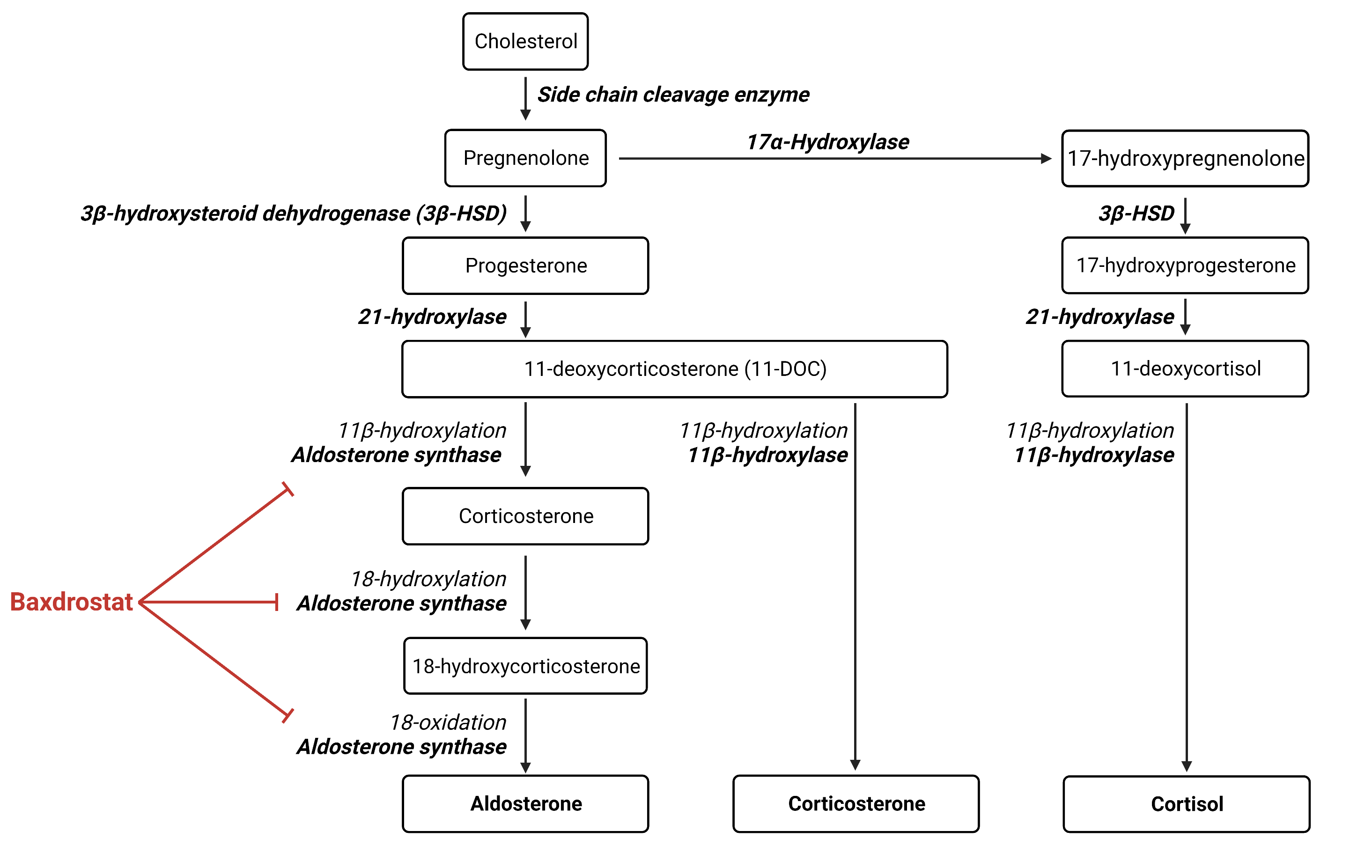


**Supplementary Figure 2. Biosynthetic Pathways for Aldosterone and Cortisol**

3β-HSD indicates 3β-hydroxysteroid dehydrogenase; 11-DOC, 11-deoxycorticosterone.
Created with BioRender.com.


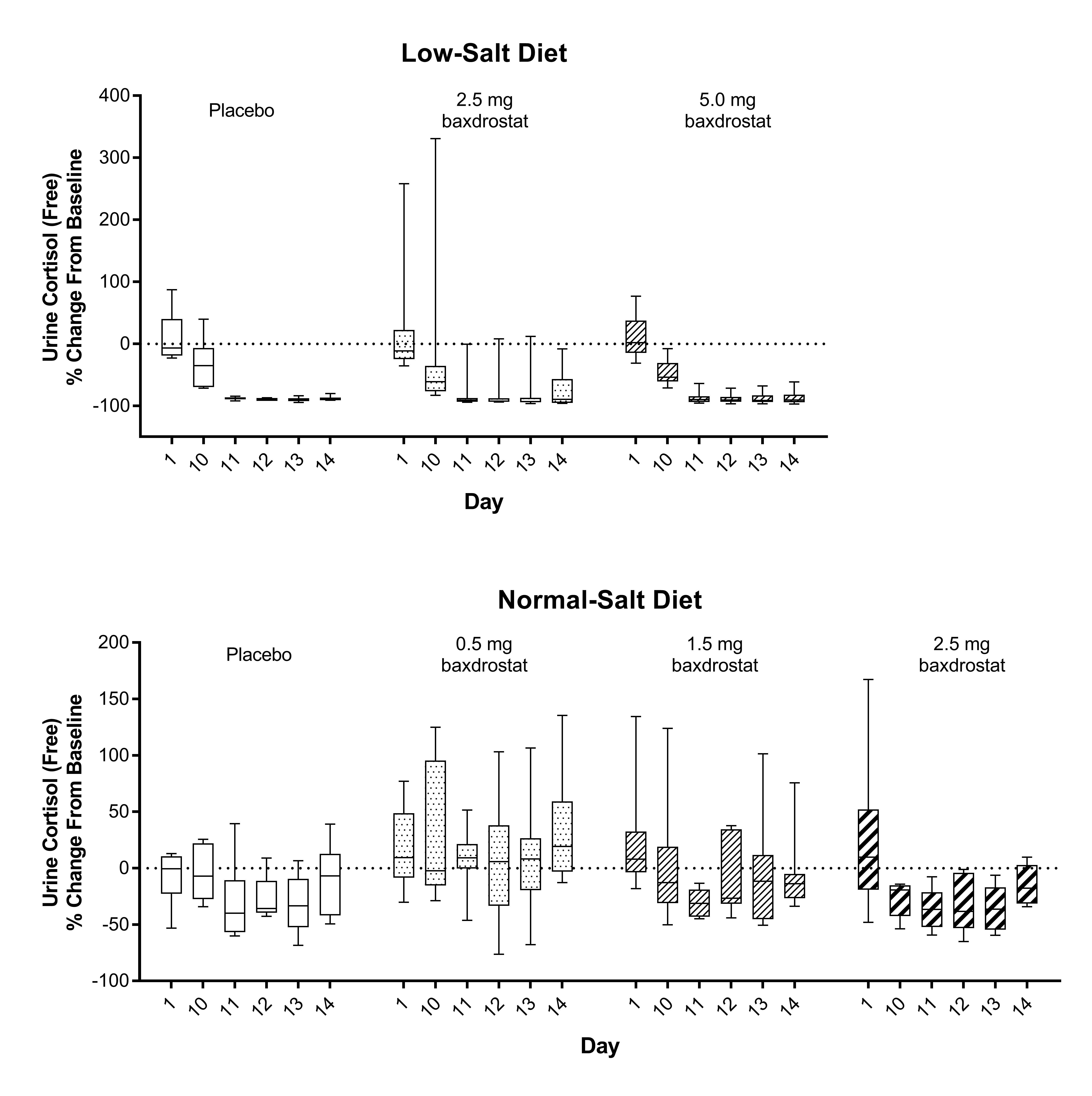


**Supplementary Figure 3. Percentage Change From Baseline in Urine Cortisol (Free) Following Baxdrostat Administration**

Urine cortisol (free) percentage change from baseline (day −1) on day 1 and day 10 through 14. Box whiskers show interquartile range (box) and minimum-maximum (whiskers) for free cortisol from 24-hour urine collections.


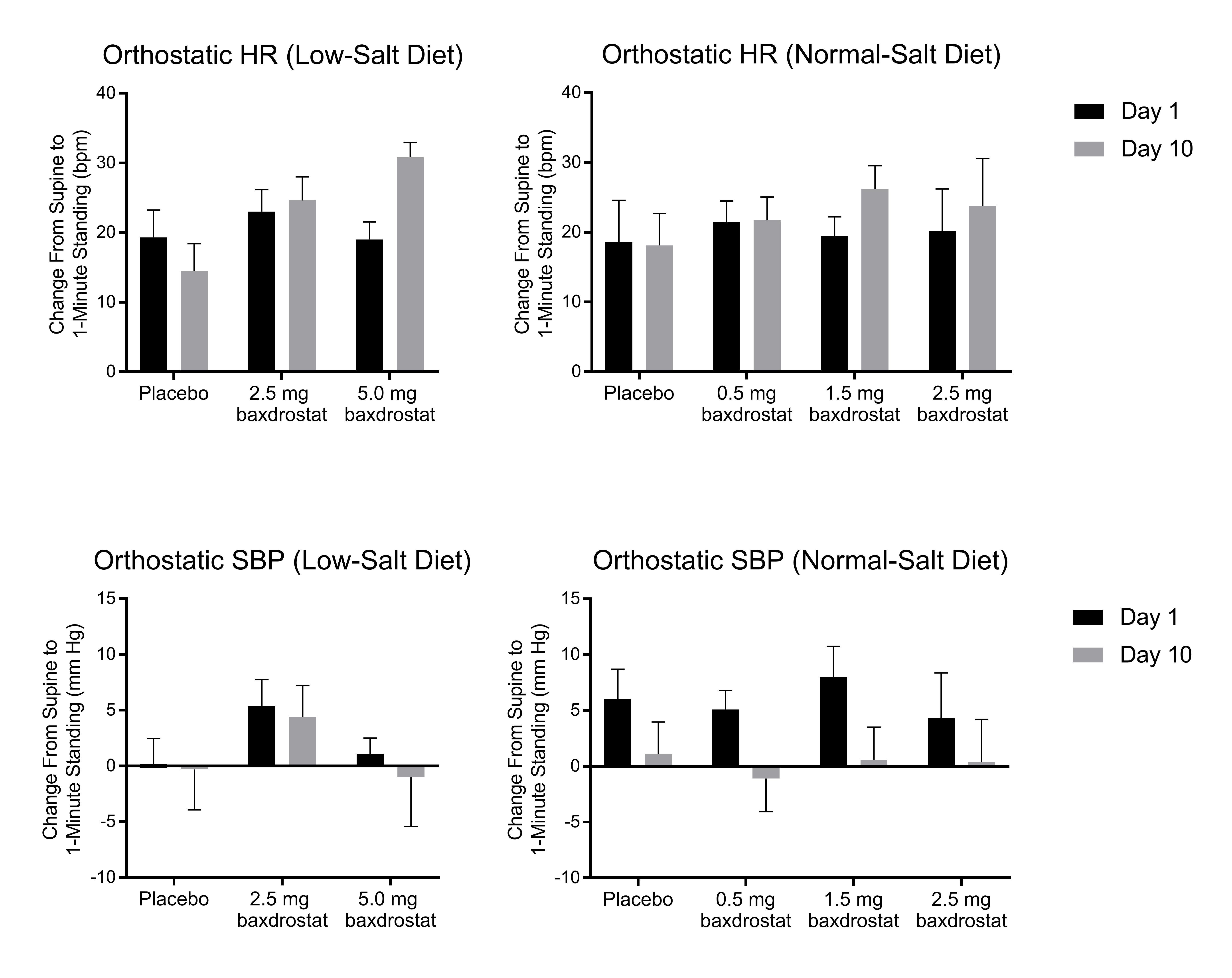


**Supplementary Figure 4. Changes in Orthostatic Blood Pressure and Heart Rate on Days 1 and 10**

Heart rate and blood pressure were measured after the subject had been in a supine position for a minimum of 5 minutes followed by standing for 1 minute. Data are mean ± standard error.

bpm indicates beats per minute; HR, heart rate; SBP, systolic blood pressure.


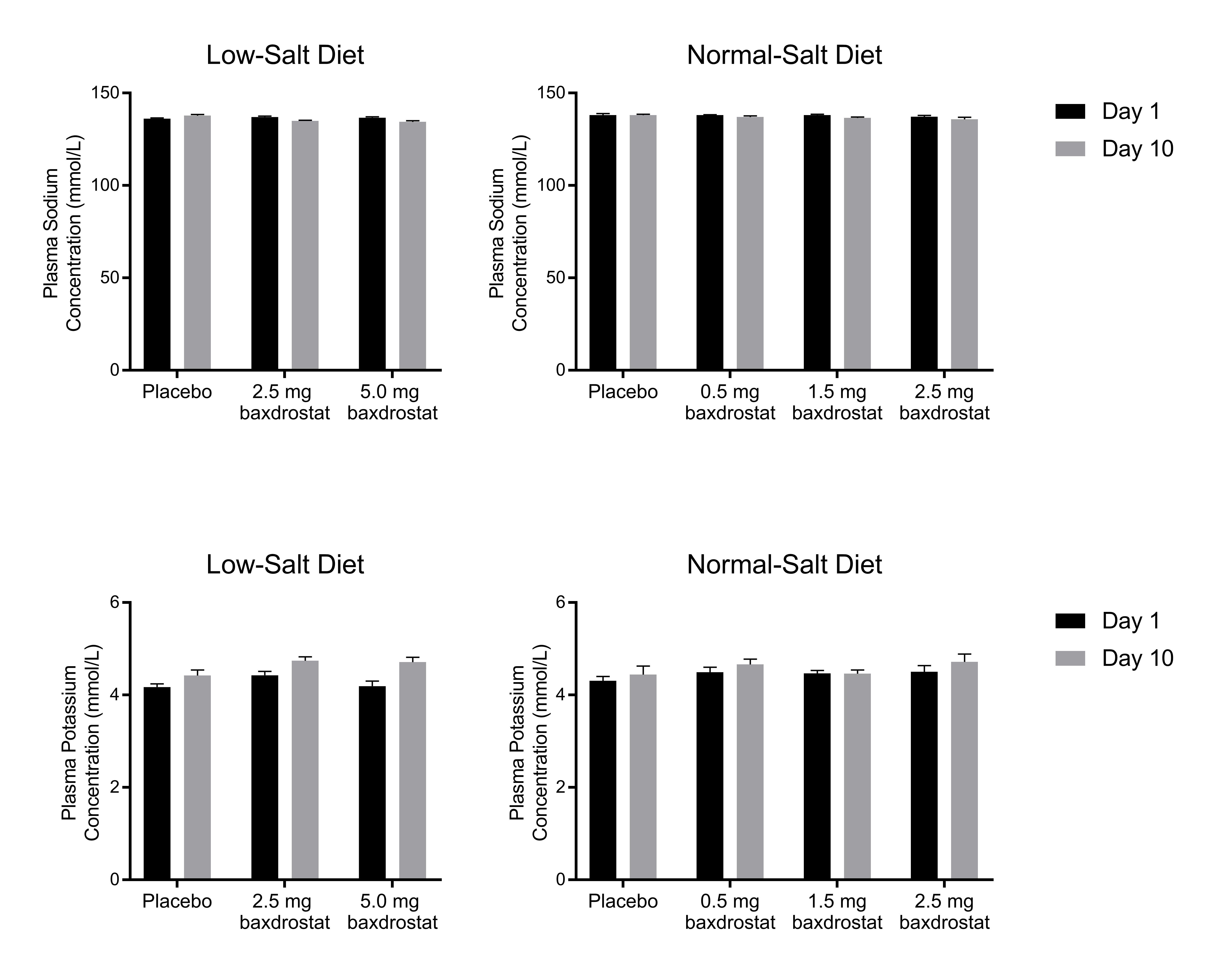


**Supplementary Figure 5. Plasma Sodium and Potassium Concentrations on Days 1 and 10**

Plasma sodium and potassium concentrations were obtained prior to baxdrostat dosing on day 1 and day 10. Data are mean ± standard error.
